# Changes in Spatial Working Memory in Stable Chronic Obstructive Pulmonary Disease: A Retrospective Study

**DOI:** 10.1155/2020/7363712

**Published:** 2020-07-22

**Authors:** Zhi Lv, Panpan Hu, Yinglin Jiang, Wanchun Yang, Rui Wang, Kai Wang, Xiaoyun Fan

**Affiliations:** ^1^Department of Geriatric Respiratory and Critical Care Medicine, The First Affiliated Hospital of Anhui Medical University, Hefei, China; ^2^Department of Pulmonary, The Second People's Hospital of Hefei (The Affiliated Hefei Hospital of Anhui Medical University), Hefei, China; ^3^Department of Neurology, The First Affiliated Hospital of Anhui Medical University, Hefei, China

## Abstract

Chronic obstructive pulmonary disease (COPD) is characterized by irreversible airflow limitation and is often accompanied by cognitive impairment. Little is known about the working memory of COPD patients. The aim of the study is to evaluate the spatial working memory of COPD patients using the classical visuospatial working memory neuropsychological paradigms. This was a retrospective study of patients with COPD who were evaluated for neurocognitive functions between February and December 2018 at Hefei Second People's Hospital. Healthy controls (HC) were included. The neuropsychological tests included the Beijing Version of the Montreal Cognitive Assessment Test (MoCA), digit span test (DS), Chinese Auditory Verbal Learning Test (CAVLT), Stroop test, and Verbal Fluency Test (VFT). The COPD group performed worse in MoCA (22.3 ± 4.5 vs. 26.1 ± 2.9, *P* < 0.001), Stroop interference test (44.2 ± 16.9 vs. 36.8 ± 10.3, *P* = 0.038), and VFT (12.9 ± 2.8 vs. 15.3 ± 4.7, *P* = 0.021) vs. the HC group. Compared with the HC group, COPD patients had statistically significant differences with respect to 0-back RT (657 ± 46 vs. 578 ± 107, *P* = 0.001), 1-back accuracy (41.8 ± 12.1% vs. 81.5 ± 18.1%, *P* < 0.001), 1-back RT (592 ± 75 vs. 431 ± 138, *P* < 0.001), 2-back accuracy (31.4 ± 9.9% vs. 68.1 ± 16.6%, *P* < 0.001), and 2-back RT (563 ± 79 vs. 455 ± 153, *P* = 0.002). Only PaO_2_ was independently associated with 0-back RT (*B* = 0.992 ± 0.428, *P* = 0.028) and 1-back ACC (*B* = 0.003 ± 0.001, *P* = 0.004). COPD patients exhibit impairment in working memory and executive function, but not in short- or long-term memory. The impairment of working memory in a patient with COPD may be more due to integrate memory information rather than to memory information storage. COPD patients exhibit a frontal-type cognitive decline.

## 1. Introduction

Chronic obstructive pulmonary disease (COPD) is a disease characterized by progressive, irreversible airflow limitation and abnormal inflammatory response in the lungs [1]. The worldwide prevalence of COPD is estimated at 4%-10% [2] and reaches 15%-47% in smokers [3]. The disease course is usually progressive, with a long-term decline in lung function, and is the third leading cause of mortality worldwide [1]. It is a preventable and treatable disease commonly associated with comorbidities (such as cardiovascular disease) and significant systemic consequences (such as skeletal muscle dysfunction) [1].

Nowadays, in addition to its physical complications, COPD is increasingly seen as a disorder that affects the brain, including anxiety, depression, and cognitive functioning [4, 5]. Patients with COPD develop cognitive impairment, either globally or in single cognitive domain such as information processing, attention and concentration, memory, executive functioning, and self-control [6–9]. In 2017, Cleutjens et al. [10] performed a full scale domain-specific cognitive function evaluation in patients with COPD and matched non-COPD controls, considering the smoking status, age, and level of education; patients with COPD had a more important proportion of cognitive impairment than the controls when considering specific cognitive domains such as psychomotor speed, planning, verbal memory, and cognitive flexibility There are several causes of cognitive impairment in COPD, including anxiety/depression, chronic hypoxic state, chronic low-grade systemic inflammation, brain damage, reduced physical activity, and exacerbations [4, 6–9].

In patients with COPD, the gray matter (GM) volume was found to be decreased in many brain regions such as the dorsolateral prefrontal cortex [11] (involved in executive functions and working memory [12]), different areas involved in visuospatial processing [13], and limbic and paralimbic structures [14] (mainly involved in emotion processing and memory). Nevertheless, little is known about the impairments in working memory in patients with COPD.

The working memory is a combined cognitive function that requires the combined functions of short-term memory and executive functions [15]. Of note, those two functions are expected to decline with age [16], which is also a risk factor for COPD [17]. The working memory is a brain system that enables temporary storage and manipulation of the information necessary for language comprehension, learning, and reasoning [18]. Some of the most common everyday behaviors involve the working memory, which is the ability to maintain and manipulate information from the outside world for a short period of time [19].

Based on the network model of working memory, specific and distinct frontal-posterior networks mediate the spatial and object working memory [20]. The dorsal areas of the prefrontal cortex (PFC) coordinate with the parietal cortex to support the spatial working memory, while the ventral areas of the PFC coordinate with the temporal cortex to support the working object memory [21]. Neuroimaging studies in humans show sustained elevated signals in the superior frontal sulcus (SFS) and posterior parietal cortex (PPC) during sustained spatial memory representations by examining delay-period functional magnetic resonance imaging (fMRI) signals [22]. Studies of patients with PFC lesions confirmed the critical role of the PFC in short-term memory and behavior [23]. In nonhuman primates, the spatial working memory depends on the highly evolved dorsolateral PFC [24].

As indicated above, COPD is a disease characterized by chronic hypoxia [4, 6–9], which will lead to brain hypoxia, including the PFC. Thus, we speculate that patients with COPD might also be with spatial working memory deficits because of this chronic hypoxia. Therefore, we used the classical visuospatial working memory neuropsychological paradigms to test the spatial working memory function in patients with COPD. We hypothesized that patients with COPD would demonstrate deficits in frontal-type cognitive functions, especially in spatial working memory.

## 2. Material and Methods

### 2.1. Study Design and Participants

This was a retrospective study of patients with COPD who were evaluated for neurocognitive functions between February and December 2018 at the Department of Respiration, Hefei Second People's Hospital, Anhui Province, China. The study was approved by the Ethics Committees of Anhui Medical University and conducted in agreement with the Declaration of Helsinki. The need for individual consent was waived by the committee.

The inclusion criteria were as follows: (1) diagnosed with COPD and classified according to the Global Initiative for Chronic Obstructive Lung Disease (GOLD) guidelines [17]; (2) stable COPD with no exacerbations during the past 8 weeks; and (3) right-handed. The exclusion criteria were as follows: (1) PaO_2_ < 60 mmHg or PaCO_2_ > 50 mmHg (breathed room air); (2) any severe comorbidities such as liver failure, kidney failure, or malignant tumor; (3) sleep apnea syndrome; (4) history of neurological or psychiatric illnesses such as depression (Hamilton Depression Rating Scale (HAMD) score ≥ 7), anxiety (Hamilton Anxiety Rating Scale (HAMA) score ≥ 7), cerebral infarction, or migraine; (5) alcohol or other substance abuse or dependence; (6) deficits in vision or hearing; (7) receiving medicines that may cause cognitive impairment; or (8) smoking within 24 h before the cognitive tests.

All patients routinely received a complete physical examination by a respiratory physician with 6 years of specialist experience. Meanwhile, arterial blood gas analysis and pulmonary function tests were performed on each patient.

Age- and education-matched healthy controls (HCs), recruited among the relatives and acquaintances of the authors, were enrolled as controls. Exclusion criteria (2)–(8) described above for the COPD group were applicable to HCs as well.

### 2.2. Neuropsychological Background Tests

All participants were routinely evaluated using neuropsychological background tests by a neuropsychologist with 10 years of specialist experience. These neuropsychological tests, including cognitive assessment, were conducted within 24 h after pulmonary function tests and arterial blood gas analysis.

The neuropsychological tests were all designed by the Hong Kong University and Anhui Medical University [25], except MoCA. The Beijing Version of the Montreal Cognitive Assessment Test (MoCA) was administered to assess global cognitive function [26]. An adjusted MoCA score < 26 was considered cognitive impairment. The digit span test (DS) and Chinese Auditory Verbal Learning Test (CAVLT) (CAVLT immediate recall) were used to measure short-term memory and long-term memory (CAVLT delay recall); higher scores indicated better short-term memory [27]. The Stroop test and Verbal Fluency Test (VFT, lists all the fruits and vegetables in 60 s) were conducted to evaluate the general executive function. For the Stroop test, higher scores indicated worse general executive function. For VFT, higher scores indicated better executive function. The HAMD and HAMA tests were used to assess the participants' symptoms of depression (score ≥ 7) and anxiety (score ≥ 7), respectively.

### 2.3. Working Memory Performance

The working memory paradigm was evaluated using a spatial *n*-back task, including 0-back, 1-back, and 2-back task blocks. The test began with four squares (boxes) shown on the screen from top to bottom and left to right. The 0-back task block was the primary task to measure attention. The advanced task, including 1-back and 2-back task blocks, was also carried out to assess the working memory performance. During the 0-back task block, participants were instructed to press the arrow key that points to the yellow box. During the 1-back, participants were instructed to press the arrow key that points to the box that was yellow last time. For example, if the uppermost box turns yellow and then the left box turns yellow, you would press the “up arrow” key because your job was to press the arrow key that points to the box that was yellow last time. During the 2-back task blocks, participants were instructed to press the arrow key that points to the box that was yellow two times ago. Each task block consisted of 20 trials. Each spatial stimulus was presented for 1500 ms with an interstimulus interval of 1500 ms. “No Response” was recorded if the individual did not press the mouse button within 3,000 ms. Before the experiment, the participants were verbally instructed and performed a practice block. Thereafter, the participants were guided to perform the tasks 0-back, 1-back, and 2-back in order. The E-Prime 1.1 software (Psychology Software Tools, Pittsburgh, PA, USA) was used to present the stimuli and to collect the accuracy and mean reaction time (RT).

### 2.4. Observational Parameters

Basic information was collected from clinical records, including age, sex, years of education, disease duration, and history of smoking and alcohol. For blood gas analysis, PaO_2_, PaCO_2_, and SaO_2_ were tested. For pulmonary function, FEV1, FVC, and FEV1/FVC were recorded. All scores of the neuropsychological tests and working memory tests mentioned above were recorded and analyzed.

### 2.5. Statistical Analysis

SPSS 17.0 (IBM, Armonk, NY, USA) was used for statistical analysis. Normal distribution and homogeneity of variance were tested. Continuous variables were tested for normal distribution using the Kolmogorov-Smirnov test, are presented as means ± standard deviations, and were analyzed using the two-sample *t*-test. Categorical data are presented as *n* (%) and were analyzed using the chi-square test or Fisher's exact test, as appropriate. Pearson's correlation analysis was performed to assess the association between clinical parameters and cognitive functions. Multiple linear regression with the stepwise method was performed to explore the potential independent factors associated with indexes of working memory. Clinically relevant factors including age, sex, years of education, disease duration, PaO_2_, PaCO_2_, and FEV1/FVC were included in the multivariable analysis. Two-sided *P* values < 0.05 were considered to be statistically significant.

## 3. Results

### 3.1. Demographic Characteristics

Thirty-seven COPD patients were initially included in the study. Eight COPD patients were excluded for respiratory failure or depression, and 29 eligible participants with 33 control subjects were finally included. The sociodemographic, clinical, depression, and anxiety characteristics of the two groups are summarized in Table 1. There were no significant differences in age, sex, education level, and depression between the COPD and HC groups. Although there were statistical differences in HAMA between the two groups (3.7 ± 1.7 vs. 2.5 ± 2.6, *P* = 0.027), neither group reached the diagnostic criteria for anxiety.

### 3.2. Background Neuropsychological Tests

As shown in Table 2, the COPD group performed significantly worse in the tests that evaluated global cognitive function (MoCA, 22.3 ± 4.5 vs. 26.1 ± 2.9, *P* < 0.001), general executive function (Stroop interference test, 44.2 ± 16.9 vs. 36.8 ± 10.3, *P* = 0.038; and VFT fruits and vegetables, 12.9 ± 2.8 vs. 15.3 ± 4.7, *P* = 0.021), compared with the HC group. There was no significant difference observed between the COPD group and the healthy control group with respect to short-term memory (digit span, CAVLT immediate recall) or long-term memory (CAVLT delay recall) (all *P* > 0.05).

### 3.3. Spatial Working Memory Test

**Figure 1:**
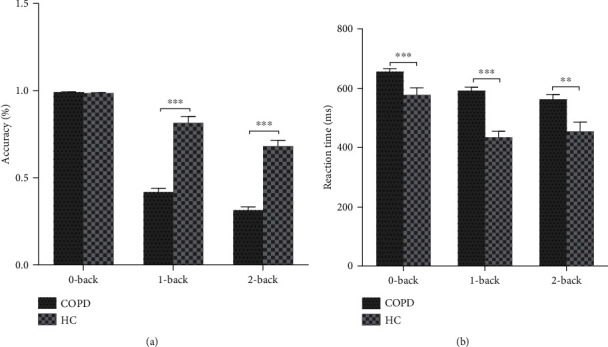
Accuracy and reaction time (RT) of working memory. (a) There were no differences between the HC and COPD groups with respect to the accuracy in the 0-back task, but there was a significant decline in the 1- and 2-back tasks. (b) Compared with the HC group, the RT was significantly longer in the COPD group in the 0-, 1-, and 2-back tasks. ^∗∗^*P* ≤ 0.01; ^∗∗∗^*P* ≤ 0.001.

Compared with the HC group, COPD patients had statistically significant differences with respect to 0-back RT (657 ± 46 vs. 578 ± 107, *P* = 0.001), 1-back accuracy (41.8 ± 12.1% vs. 81.5 ± 18.1%, *P* < 0.001), 1-back RT (592 ± 75 vs. 431 ± 138, *P* < 0.001), 2-back accuracy (31.4 ± 9.9% vs. 68.1 ± 16.6%, *P* < 0.001), and 2-back RT (563 ± 79 vs. 455 ± 153, *P* = 0.002) (Table 2 and Figure 1).

### 3.4. Correlations between Clinical Parameters and Cognitive Function

**Figure 2:**
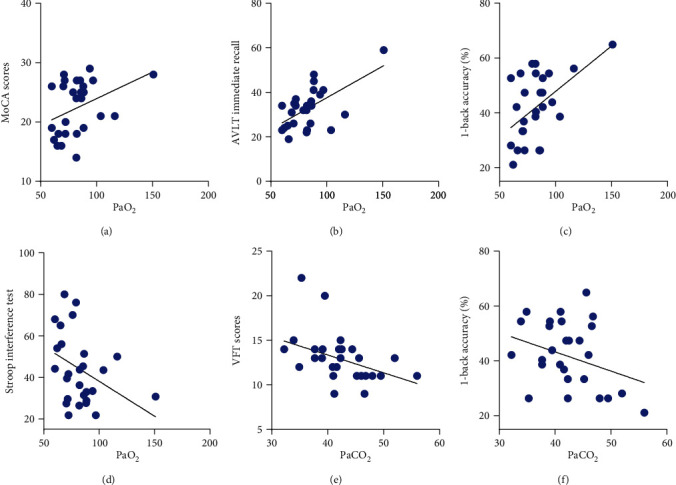
Correlation analysis between PaO_2_, PaCO_2_, and cognitive functions. (a–c) There were positive correlations between PaO_2_ and MoCA (*r* = 0.375, *P* = 0.045), immediate recall (*r* = 0.602, *P* = 0.001), and 1-back accuracy (*r* = 0.517, *P* = 0.004) in the COPD group. (d) There was a negative correlation between PaO_2_ and Stroop test (*r* = −0.375, *P* = 0.045) (longer reaction time means poorer executive function). (e, f) There were negative correlations between PaCO_2_ and VFT (*r* = −0.397, *P* = 0.033) and 1-back accuracy (*r* = −0.404, *P* = 0.030).

Pearson's correlation analysis showed that there were correlations between PaO_2_ with MoCA (*r* = 0.375, *P* = 0.045), CAVLT immediate recall (*r* = 0.602, *P* = 0.001), Stroop interference test (*r* = −0.375, *P* = 0.045), the RT in the 0-back task (*r* = 0.407, *P* = 0.028), and the accuracy in the 1-back task (*r* = 0.517, *P* = 0.004), but not in CAVLT delay recall (*r* = 0.354, *P* = 0.059), VFT (*r* = 0.194, *P* = 0.314), the accuracy in the 0-back task (*r* = 0.040, *P* = 0.838), the RT in the 1-back task (*r* = −0.005, *P* = 0.978), the accuracy in the 2-back task (*r* = 0.114, *P* = 0.557), and the RT in the 2-back task (*r* = −0.193, *P* = 0.316) in the COPD group. There was a negative correlation between PaCO_2_ and VFT (*r* = −0.397, *P* = 0.033) and between PaCO_2_ and 1-back ACC (*r* = −0.404, *P* = 0.030), but no significant correlations between PaCO_2_ and any other cognitive functions (all *P* > 0.05). There were no significant correlations between FEV1/FVC and any cognitive functions in the COPD group (all *P* > 0.05) (Table 3 and Figure 2).

### 3.5. Multivariable Analysis of Working Memory

Only PaO_2_ was independently associated with 0-back RT (*B* = 0.992 ± 0.428, *P* = 0.028) and 1-back ACC (*B* = 0.003 ± 0.001, *P* = 0.004).

## 4. Discussion

COPD is characterized by irreversible airflow limitation [1] and chronic systemic hypoxia [4, 6–9] and is often accompanied by cognitive impairment [4]. On the other hand, little is known about the working memory of patients with COPD. Therefore, this study is aimed at evaluating the spatial working memory of patients with COPD using the classical visuospatial working memory neuropsychological paradigms. The results suggest that patients with COPD had impairments in working memory and executive function, but not in short- or long-term memory. The impairment of the working memory in a patient with COPD may be more due to integrating memory information rather than memory information storage. Patients with COPD exhibit a frontal-type cognitive decline.

COPD is a lung disease with remarkable cognitive impairments as extrapulmonary features, and COPD is even considered a cognitive-pulmonary disease [7]. As a feature of the pulmonary-type cognitive impairment, patients with COPD performed significantly worse in the tests that evaluated global cognitive function (i.e., the MoCA) and executive function, compared with HCs. In addition, patients with COPD showed no significant memory deficits in either short- or long-term memory. The occurrence of cognitive impairment in patients with COPD varies among studies from 12% to 88% [28], depending upon hypoxia, exacerbation, vascular risk factors, and the severity of COPD [8]. The prevalence of general cognitive impairment in COPD was 5.5% in a large sample of adults with COPD, measured with the Mini-Mental State Examination (MMSE) [29]. Compared with the MMSE, the MoCA scale has a higher sensitivity for the diagnosis of cognitive dysfunction [30]. Increasing age is the most significant determinant of cognitive impairment [31]. Cross-sectional and longitudinal studies showed that across cognitive domains, memory performance declines with increasing age [32]. Researchers used the Visual Verbal Learning Test, as in the present study, and found that the mean of the total immediate recall score was 40.5 vs. 32.4 in the COPD group and 46.7 vs. 32.7 in the HC group, with a mean age of 63.7 vs. 72.9 years and 60.3 vs. 72.9 years, respectively [10]. Another study, comparing the cognitive functions of Alzheimer's disease (AD) with and without COPD, found that AD-COPD had worse outcomes in executive functions screening than AD-only, but no significant differences were found in memory function [33]. Thus, we speculate that there is an age-related floor effect in the memory domain, but not in the executive function. Executive dysfunction may be a characteristic indicator to identify cognitive impairment of COPD. Of course, this will have to be examined in future studies.

In the present study, the *n*-back tasks were selected to investigate the working memory in patients with COPD. The results strongly suggest that patients with COPD had working memory impairments, either in accuracy or in RT. In the *n*-back task, the 0-back accuracy was higher in the COPD group than in HCs, but the difference was not statistically significant. On the other hand, the RT was significantly longer in the COPD group than in HCs. The 0-back task block is the primary task to measure attention [25]. Albeit nonsignificant, this higher performance of patients with COPD in the 0-back task could be due to neglect in reaction time in pursuit of high accuracy. The higher accuracy also showed that patients with COPD could concentrate on the test well but with poor process speed. The working memory is a system that includes maintaining, monitoring, and manipulating information in short-term memory, which especially needs the involvement of the dorsolateral PFC [12]. Structural MRI studies found decreased GM in the dorsolateral PFC [11], and this part of the brain is involved in executive functions and working memory [12]. Resting-state fMRI showed that patients with COPD have lower functional connectivity values in the cingulate cortex and PFC [34]. PFC plays an essential role in executive function, which is considered the higher cognitive function that would be more often affected. We found that the executive function was lower both in the Stroop test and VFT, but memory differences were not found between the COPD and HC groups.

In accordance with the brain hypoxia hypothesis, correlation analysis between clinical parameters and cognitive functions indicates that PaO_2_ is correlated with most of the cognitive function indexes such as MoCA, immediate memory, executive function, and working memory in the COPD group. There are numerous studies showing the correlation between cognitive functioning and hypoxia [35]. Patients with COPD and hypoxia have more cognitive impairments than patients with COPD without hypoxia [36]. Patients with COPD and long-term oxygen therapy exhibit a significant decrease in cognitive status compared with patients with COPD without regular-use of long-term oxygen therapy [37]. A SPECT study showed that anterior cerebral hypoperfusion and selected neuropsychological dysfunctions were characteristics of hypoxemic patients with COPD and could indicate frontal-type cognitive decline in patients with worsening hypoxemia [38].

For a long time, the relationship between hypercapnia, pulmonary function, and cognitive functions has been controversial. Hypercapnia plays a less essential role in cognitive function compared with hypoxia [10]. The present study indicated that there was a negative correlation between PaCO_2_ and executive function (VFT), but no significant correlations were observed between PaCO_2_ and the other indicators of cognitive functions. Cognitive impairment in patients with severe to very severe COPD seems to be associated with the severity of airway obstruction [36], but not all studies show a relationship between FEV1 and cognitive functioning [7]. In the present study, there was a negative correlation between FEV1 with the accuracy in the 2-back task, but no significant correlations between FEV1 with any other cognitive function indexes in the COPD group. This warrants a further study.

Several limitations of the present study should be taken into consideration. First, the sample size was small, and the severity of the disease was not considered. Future studies should include participants with different severities of COPD to assess working memory. Second, MRI/CT scan was not performed to rule out small vascular diseases and other asymptomatic brain damage such as lacunar infarction. An MRI scan is necessary for further studies to explore the brain structure and functional connectivity in both COPD and HC participants. Third, the working memory included spatial and object working memory. Although spatial-based working memory impairment was more usual for COPD patients, object-based working memory has not been yet clarified. We need to assess whether there are changes in object working memory in COPD patients. Finally, the MOCA scores of control patients were 19-30 points, indicating mild cognitive impairment in some individuals, which is not surprising for this age group.

## 5. Conclusion

In conclusion, COPD patients exhibited significant impairment in working memory and executive function, but not in short- or long-term memory. The impairment of working memory in patients with COPD may be due to the integration of memory information rather than the storage of memory information. The cognitive decline did not occur equally across all cognitive domains in patients with COPD and HCs. Patients with COPD exhibited a frontal-type cognitive decline.

## Figures and Tables

**Table 1:** Demographic characteristics of the participants.

Characteristics	COPD (*n* = 29)	HC (*n* = 33)	*P*
Age (years), mean ± SD	72.9 ± 5.6	72.9 ± 4.5	0.992
Sex (male), *n* (%)	26 (89.7)	25 (75.8)	0.153
Education (years), mean ± SD	6.2 ± 2.7	6.1 ± 4.1	0.927
Disease duration (years), mean ± SD	9.0 ± 6.6	NA	
History of smoking, *n* (%)	26 (89.7)	NA	
History of alcohol, *n* (%)	9 (31.0)	NA	
Blood gas analysis, mean ± SD			
PaO_2_ (mmHg)	81.7 ± 18.9	NA	
PaCO_2_ (mmHg)	42.1 ± 5.5	NA	
SaO_2_ (%)	94.4 ± 2.1	NA	
Pulmonary function, mean ± SD			
FEV1 (L/sec)	1.0 ± 0.4	NA	
FVC (L)	1.9 ± 0.7	NA	
FEV1/FVC (%)	53.6 ± 8.3	NA	
HAMA, mean ± *S*D	3.7 ± 1.7	2.5 ± 2.6	0.027
HAMD, mean ± SD	2.7 ± 2.0	1.7 ± 2.2	0.078

PaO_2_: arterial oxygen tension; PaCO_2_: arterial carbon dioxide tension; SaO_2_: arterial oxygen saturation; FEV1: forced expiratory volume in one second; FVC: forced vital capacity; FEV1/FVC: forced vital capacity/forced expiratory volume in one second. HAMA: Hamilton anxiety rating scale; HAMD: Hamilton depression rating scale; COPD: chronic obstructive pulmonary disease; HC: healthy control; NA: not applicable.

**Table 2:** Neuropsychological tests and working memory performance.

Variables, mean ± *SD*	COPD (*n* = 29)	HC (*n* = 33)	*P*
Global cognitive function			
MoCA	22.3 ± 4.5	26.1 ± 2.9	<0.001
Short term memory			
DS-forward	6.0 ± 1.7	6.2 ± 1.4	0.702
CAVLT immediate recall	32.4 ± 8.9	32.7 ± 11.3	0.914
Long term memory			
CAVLT delay recall	6.1 ± 3.4	7.2 ± 2.6	0.127
Executive function			
Stroop interference test (sec)	44.2 ± 16.9	36.8 ± 10.3	0.038
VFT fruits and vegetables	12.9 ± 2.8	15.3 ± 4.7	0.021
Working memory			
Primary task			
0-back ACC (%)	99.1 ± 1.2	98.6 ± 1.5	0.145
0-back RT (ms)	657 ± 46	579 ± 107	0.001
Advanced task			
1-back ACC (%)	41.8 ± 1219	81.5 ± 18.1	<0.001
1-back RT (ms)	592 ± 75	431 ± 138	<0.001
2-back ACC (%)	31.4 ± 9.9	68.1 ± 16.6	<0.001
2-back RT (ms)	563 ± 79	455 ± 153	0.002

MoCA: Montreal Cognitive Assessment Test; DS: digit span test; CAVLT: Chinese Auditory Verbal Learning Test; VFT: Verbal Fluency Test; ACC: accuracy; RT: reaction time.

**Table 3:** Correlation analysis between clinical parameter and cognitive functions of COPD patients.

	PaO_2_		PaCO_2_		FEV1/FVC	
	*r*	*P*	*r*	*P*	*r*	*P*
Global cognitive function						
MoCA	0.375	0.045	-0.185	0.336	0.183	0.341
Short term memory						
DS-forward	0.238	0.156	-0.044	0.798	0.075	0.658
AVLT immediate recall	0.602	0.001	-0.143	0.461	-0.121	0.531
Long term memory						
AVLT delay recall	0.354	0.059	-0.064	0.743	0.126	0.514
Executive function						
Stroop interference test	-0.375	0.045	0.160	0.407	0.015	0.939
VFT fruits and vegetables	0.194	0.314	-0.397	0.033	0.183	0.343
Working memory						
Primary task						
0-back ACC	0.040	0.838	0.138	0.476	-0.012	0.949
0-back RT	0.407	0.028	-0.213	0.268	-0.209	0.278
Advanced task						
1-back ACC	0.517	0.004	-0.404	0.030	-0.083	0.670
1-back RT	-0.005	0.978	-0.073	0.707	0.029	0.882
2-back ACC	0.114	0.557	-0.208	0.278	0.094	0.628
2-back RT	-0.193	0.316	-0.105	0.586	0.133	0.490

MoCA: Montreal Cognitive Assessment Test; DS: digit span test; CAVLT: Chinese Auditory Verbal Learning Test; VFT: Verbal Fluency Test; ACC: accuracy; RT: reaction time.

## Data Availability

The datasets used and/or analyzed during the current study are available from the corresponding author on reasonable request.
